# Implementation of the WHO IPC Ring Approach During the 2022 Uganda Sudan Ebola Virus Disease (SUDV) Response at the Epicentre

**DOI:** 10.1017/ash.2024.286

**Published:** 2024-09-16

**Authors:** Maureen Kesande, Elizabeth Katwesigye, Judith Nanyondo, Shillah Nakato, Privato Ainembabazi, Resty Nanyonjo, Mary Namusoke, Doreen Nabawanuka, April Baller

**Affiliations:** Infectious Diseases Institute, Uganda; Ministry of Health, Uganda; Infectious Diseases Institute, Makerere University; World Health Organization

## Abstract

**Background:** At the onset of an outbreak, immediate infection, prevention and control (IPC) measures and strategies are critically important in stopping the transmission. During the 2022 Sudan Virus Disease (SUDV) outbreak in Uganda, the IPC technical working group (TWG) adopted the WHO ring approach for intensive and targeted IPC support to interrupt transmission in high-risk areas and healthcare facilities (HCFs). Objectives: a) Leverage surveillance and epidemiological activities to guide response efforts and implement targeted IPC interventions. b) To rapidly interrupt SUDV transmission at the source through multiple IPC interventions. **Methods:** The IPC TWG delineated outbreak perimeters (rings) to include health facilities and community sites within 500 meters in urban centres and 1 kilometer in rural areas around each confirmed case. A data base with this information was developed and updated daily with information provided by the surveillance team. To activate response within 12 hours, interventions included rapid needs and risk assessments , health educational materials, deployment of decontamination teams and district IPC mentors with hygiene supplies delivered within 24 hours and a 72-hour follow-up. Trained Village Task Forces (VTF) and IPC mentors conducted health education, set up screening points, holding units, and rapid notification channels. **Results:** 56 rings including HCFs (38) and community sites (78) were identified within the radius of confirmed cases. Using the IPC scorecard, health facility mean scores significantly increased from 18% to 61.7% at follow-up in three weeks. Community WASH baseline scores improved from 11.1% (inadequate) to 69% with a basic level in two weeks. There was marked reduction in the incidence of new cases in the epicentre within the first 32 days. **Conclusion:** The results suggest that the IPC ring approach is an instrumental strategy health ministries can adopt to rapidly provide targeted comprehensive support at the source to interrupt transmission. A collaborative effort across pillars and partners in the implementation of the ring approach is key through concerted efforts and information sharing across response pillars.

